# How SUMOylation Fine-Tunes the Fanconi Anemia DNA Repair Pathway

**DOI:** 10.3389/fgene.2016.00061

**Published:** 2016-04-19

**Authors:** Kate E. Coleman, Tony T. Huang

**Affiliations:** Department of Biochemistry and Molecular Pharmacology, New York University School of Medicine, New YorkNY, USA

**Keywords:** Fanconi anemia (FA), SUMOylation, SUMO-targeted ubiquitin ligases (STUbLs), SUMO proteases (SENPs), SUMO-interaction motifs (SIMs), SUMO-like domains (SLDs), deubiquitinases (DUBs), DNA interstrand crosslinks (ICLs)

## Abstract

Fanconi anemia (FA) is a rare human genetic disorder characterized by developmental defects, bone marrow failure and cancer predisposition, primarily due to a deficiency in the repair of DNA interstrand crosslinks (ICLs). ICL repair through the FA DNA repair pathway is a complicated multi-step process, involving at least 19 FANC proteins and coordination of multiple DNA repair activities, including homologous recombination, nucleotide excision repair and translesion synthesis (TLS). SUMOylation is a critical regulator of several DNA repair pathways, however, the role of this modification in controlling the FA pathway is poorly understood. Here, we summarize recent advances in the fine-tuning of the FA pathway by small ubiquitin-like modifier (SUMO)-targeted ubiquitin ligases (STUbLs) and other SUMO-related interactions, and discuss the implications of these findings in the design of novel therapeutics for alleviating FA-associated condition, including cancer.

## Fanconi Anemia (FA) DNA Repair Pathway

Fanconi anemia is a very rare autosomal recessive disease (occurring in just 1 of every 100,000 births), typically distinguished by bone marrow abnormalities, cancer predisposition, and congenital anomalies (Kee and D’Andrea, 2012). Patients with this disease exhibit defects in the repair of interstrand crosslinks (ICLs), DNA lesions which hook bases of opposing DNA strands together, and are consequently hypersensitive to crosslinking agents such as cisplatin, diepoxybutane (DEB), and mitomycin C (MMC) (Noll et al., 2006). Despite the low prevalence of the FA disease, the complex pathway involved in the recognition and repair of ICLs orchestrates multiple DNA repair processes, such as homologous recombination (HR) and translesion synthesis (TLS), making FA an important model in the study of DNA repair signaling.

To date, 19 FANC proteins have been identified in the FA pathway, which fall into three distinct functional groups. One group is the FA core complex, consisting of FANCA, B, C, E, F, G, L, and M, which together with three Fanconi associated proteins (FAAP20, FAAP24, and FAAP100) functions as an E3 ubiquitin ligase (Garcia-Higuera et al., 2001; Meetei et al., 2003; Ciccia et al., 2007; Ling et al., 2007; Leung et al., 2012). Upon pathway induction by DNA damage or replication stress, the targeting components of the FA core, FANCM and FAAP24, bind chromatin and recruit the FA core complex (Qiao et al., 2001; Kim et al., 2008). A histone-fold-containing complex consisting of MHF1-MHF2 proteins facilitates the recruitment of FANCM to chromatin to enhance pathway activation (Singh et al., 2010; Yan et al., 2010). The FA core complex subsequently monoubiquitylates group II proteins, FANC**I** and FANC**D2**, which associate to form a heterodimer called the ID complex (Garcia-Higuera et al., 2001; Sims et al., 2007; Smogorzewska et al., 2007). This monoubiquitylation of the ID complex localizes it to chromatin, where it recruits the group III effector proteins to initiate downstream ICL repair.

**FIGURE 1:**
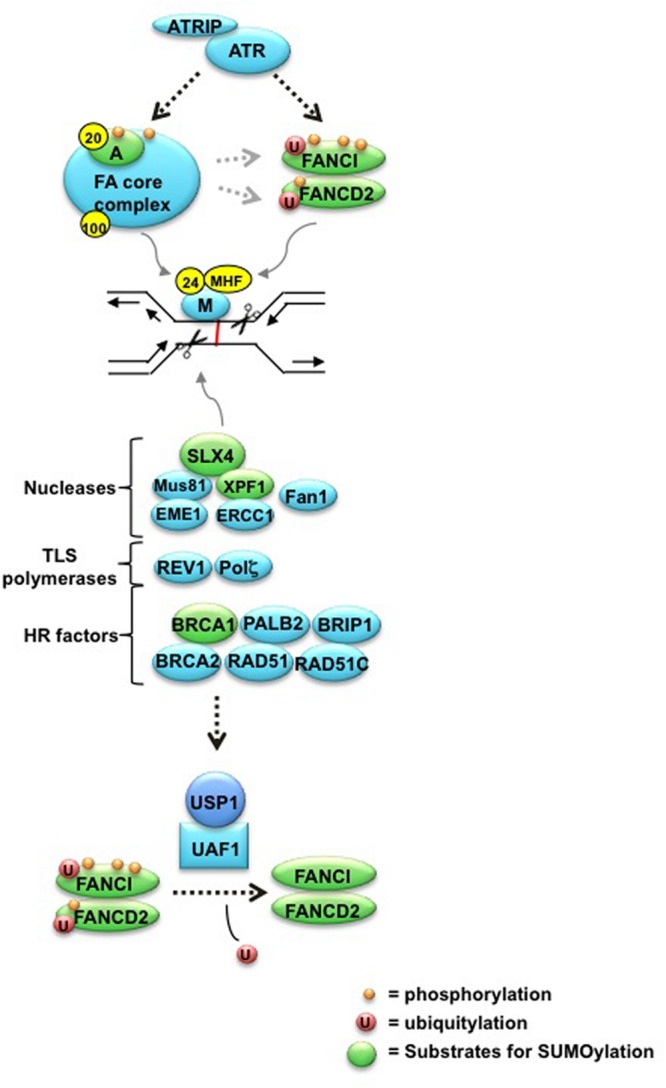
**The ICL repair pathway.** The FANCM-FAAP24 complex first recognizes stalled replication structures, and recruits the FA core complex to the ICL. ATR-Chk1 phosphorylates and activates multiple components, including FANCA, E, I, and D2, which is required for monoubiquitylation of the ID complex by the FA core complex. Upon monoubiquitylation, the activated ID complex localizes to chromatin and coordinates the activities of downstream nucleases that create incisions around the crosslink. The resulting structure is then repaired by multiple processes, involving lesion bypass by TLS polymerases, HR, and NER. Finally, the USP1-UAF1 DUB complex removes monoubiquitin from the ID complex to complete repair. See accompanying text for more details. Yellow spheres indicate Fanconi Anemia-associated proteins (FAAPs) FAAP20, FAAP24, and FAAP100. Targets for SUMOylation highlighted in this review are denoted in green.

Following ID monoubiquitylation, a complex series of steps ensues to complete ICL repair. First, the mono-ubiquitinated ID complex serves as a scaffold for recruitment of several nucleases, which make nucleolytic incisions flanking the ICL to unhook the crosslink. These nucleases include SLX4 (discussed in more detail below), ERCC1-XPF1, and MUS81-EME1 structure-specific endonucleases (Hanada et al., 2006; Knipscheer et al., 2009; Stoepker et al., 2011; Klein Douwel et al., 2014). Cross-linked nucleotides on the complementary strand are subsequently bypassed by the process of TLS, which involves specialized Y-family polymerases such as polζ and REV1 (Räschle et al., 2008; Kim et al., 2012; Budzowska et al., 2015). The nucleolytic incisions result in a double strand break (DSB) in the DNA, which is repaired by HR (discussed below), and nucleotide excision repair (NER) fills the remaining gap (Räschle et al., 2008; Kim and D’Andrea, 2012). Finally, in the last step, the deubiquitinating (DUB) complex USP1-UAF1 removes monoubiquitin from the ID complex, allowing for pathway regeneration (Nijman et al., 2005; Cohn et al., 2007) (**Figure 1**). For more extensive reviews on the steps of the FA pathway, please refer to the following reviews: (Kim and D’Andrea, 2012; Walden and Deans, 2014; Duxin and Walter, 2015). Despite extensive study, it is likely that additional aspects of FA pathway activation and regulation remain unidentified, prompting recent studies of other regulatory proteins and post-translational modifications (PTMs) involved in the FA pathway.

## Control of Signaling Pathways By SUMOylation

In addition to ubiquitin signaling, modification of proteins by small ubiquitin-like modifier (SUMO) has been implicated in several aspects of cellular signaling. Similar to protein ubiquitylation, the process of activating and conjugating SUMO modifications to substrates involves an E1-E2-E3 enzyme cascade. SUMO proteins are initially translated as immature precursors that must be processed by proteases to a mature form containing a C-terminal diglycine motif. This mature form of SUMO subsequently becomes bound by an E1 enzyme that activates SUMO through sequential adenylation and thioester bond formation. SUMO is then passed to a single E2 conjugating enzyme, ubiquitin-like conjugating enzyme 9 (Ubc9), that transfers the SUMO to substrate accepter lysine residues with the help of E3 protein ligases. Protein SUMOylation is mediated by several SUMO E3 ligases in mammalian cells, including those of the PIAS family: PIAS1, PIAS2 (PIASxα/β), PIAS3, and PIAS4 (PIASy). These E3 enzymes facilitate SUMO conjugation either by promoting specificity to substrate recruitment by E2∼SUMO, or by stimulating the discharge of SUMO to substrates (Gareau and Lima, 2010). The E2 enzyme Ubc9 recognizes its substrates through the consensus SUMOylation motif, ΨKxE/D, where Ψ is a large hydrophobic residue (Rodriguez et al., 2001). Conversely, SUMO can also be de-conjugated from substrates through the activity of SUMO specific proteases (SENPs), making this a highly dynamic modification analogous to ubiquitylation and phosphorylation (Kumar and Zhang, 2015).

Another family of proteins called SUMO-targeted ubiquitin ligases (STUbLs) has also been characterized recently that connects the processes of SUMOylation with ubiquitylation. Uniquely, STUbL enzymes recognize their substrates through SUMO-interaction motifs (SIMs), short hydrophobic peptide sequences that mediate non-covalent attachments with SUMO (Perry et al., 2008). Often, STUbL enzymes promote the specific recruitment of proteins containing both ubiquitin-interacting motifs (UIMs) and SIMs through the synthesis of SUMO-ubiquitin conjugate chains. As an example, the RAP80 subunit of the BRCA1 complex uses both its SIM and UIM motifs to bind SUMO-ubiquitin conjugate chains synthesized by the STUbL RNF4 for efficient recruitment to DSBs immediately following DNA damage (Guzzo et al., 2012; Hu et al., 2012). By promoting the degradation of previously SUMOylated target proteins, STUbL enzymes play important roles as global regulators of SUMOylation levels. Imbalances in global protein SUMOylation levels can have several adverse cellular consequences, including genome instability and sensitivity to genotoxic stress (Perry et al., 2008).

Small ubiquitin-like modifier and SUMO-like modifications can influence a wide variety of cellular signaling pathways by directing changes in protein-protein interactions, altering protein intracellular localization, directing protein turnover (via STUbL enzymes described above), or changing protein activity. Despite its importance in these other signaling pathways (reviewed in Gareau and Lima, 2010), a functional role for ubiquitin-SUMO crosstalk in the Fanconi Anemia pathway has not been defined until recently. This review highlights new evidence of FA pathway modulation by SUMO modifications and SUMO-like interactions, and describes the impact of these observations on our understanding of FA pathway regulation and disease treatment.

## Role of FANCA SUMOylation

**FIGURE 2:**
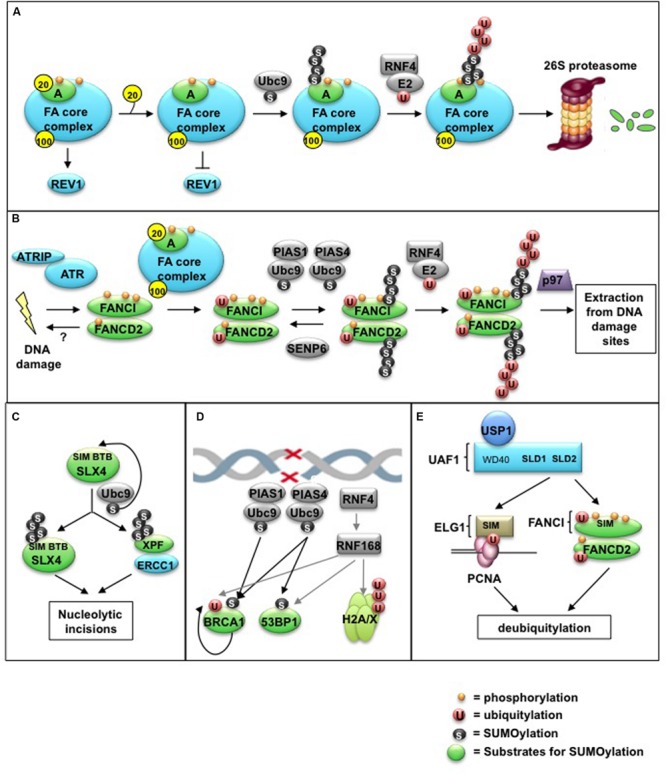
**Control of the FA pathway by SUMO and SUMO-like modifications. (A)** Regulated release of the FAAP20 subunit from FANCA is required for SUMOylation of FANCA via the E2 conjugating enzyme Ubc9. Removal of FAAP20 inhibits the recruitment of the downstream TLS polymerase REV1 during ICL repair, resulting in decreased TLS-mediated mutagenesis. SUMOylation of FANCA subsequently triggers RNF4-mediated polyubiquitylation and proteasome-mediated degradation. **(B)** The ID complex is targeted for SUMOylation in a manner dependent on the activities of the ATR kinase, the FA core complex, and the SUMO E3 ligases PIAS1/PIAS4. Alternatively, SUMOylation can be removed by the SUMO protease SENP6. SUMOylation of the ID complex allows for recognition by RNF4, leading to ID complex polyubiquitylation and removal from damage sites via the DVC1-p97 segregase complex. **(C)** The SLX4 complex acts as a SUMO E3 ligase by triggering its own SUMOylation in addition to that of the DNA repair/recombination endonuclease XPF. This SLX4-dependent SUMOylation is dependent on the E2 Ubc9, as well as its own SIMs and BTB domains, and is important to prevent mitotic catastrophe following CFS expression. **(D)** The SUMO E3 ligases PIAS1 and PIAS4 promote DSB repair by localizing to damage sites and SUMOylating multiple target proteins, including BRCA1 and 53BP1. This SUMOylation is required for proper ubiquitin-adduct formation mediated by RNF8, RNF168, and BRCA1 to initiate a DNA damage response (DDR). **(E)** The SUMO-like domain SLD2 of UAF1 directs USP1-UAF1 targeting to its substrates via SIMs located within FANCI and the PCNA-interacting protein hELG1. These SLD-SIM interactions regulate deubiquitylation of FANCD2 and PCNA-Ub substrates, respectively, to coordinate HR and TLS activities during FA DNA repair.

One example of how SUMOylation contributes to FA pathway regulation is through promoting the polyubiquitylation of FA core complex member FANCA. A recent study from the D’Andrea lab identified a patient with a point mutation in FANCA (FANCA^I935S^) that fails to bind the FAAP20 subunit of the FA core complex, leading to decreased FANCA protein levels. In uncovering the mechanism behind this decreased stability of FANCA, the authors discovered that defective FAAP20 binding by the FANCA mutant leads to increased exposure of a SUMOylation site on FANCA at residue K921, which in turn promotes UBC9-mediated SUMOylation, polyubiquitylation by the STUbL RNF4, and proteasome-dependent degradation of FANCA. Failure of the FANCA mutant to bind FAAP20 still allows efficient FANCD2 monoubiquitylation, but leads to an inability to properly recruit the REV1 translesion polymerase (Kim et al., 2012), contributing to lower rates of TLS-mediated mutagenesis. On the other hand, the authors showed that wild-type FANCA is also subject to SUMOylation and RNF4-mediated polyubiquitylation, but to a lesser degree, and depletion of RNF4 contributed to increased MMC sensitivity (Xie et al., 2015) (**Figure 2A**). Taken together, these results indicate that the regulated release of FAAP20 from FANCA is a normal critical step in the FA pathway and suggest that failure to properly release FANCA from the FA core complex could contribute to pathway disruption and genome instability.

## SUMOylation of the ID Complex for the FA Pathway

As discussed previously, ID complex monoubiquitylation is removed by the USP1-UAF1 (WDR48) DUB complex, allowing for FA pathway regeneration (Nijman et al., 2005; Cohn et al., 2007). Evidence shows that depletion of USP1 in either murine models or chicken DT40 cells enhances chromatin loading of the ID complex in the absence of exogenous DNA damage (Oestergaard et al., 2007; Kim et al., 2009), but only to similar levels as observed following mitomycin C (MMC) treatment, suggesting the existence of alternative mechanisms to restrain ID complex loading at DNA lesions. Work by Gibbs-Seymour et al. (2015) demonstrates that direct SUMOylation of the ID complex can also stimulate the removal of activated ID complexes at sites of damage, thereby controlling ID complex dosage at DNA lesions. ID complex SUMOylation is dependent on the ATR kinase and two SUMO E3 ligases, PIAS1 and PIAS4, and is antagonized by the SUMO protease SENP6. Following SUMOylation, the STUbL RNF4 targets the ID complex for polyubiquitylation, ultimately promoting ID complex chromatin extraction from DNA lesions by the DVC1-p97 ubiquitin segregase complex. This ubiquitin-SUMO network thereby helps fine-tune ID complex recruitment to DNA damage sites. The authors further show that dysregulation of this process through expression of a SUMOylation-deficient FANCI mutant results in increased DNA damage and MMC sensitivity, illustrating the importance of this mechanism for limiting ID complex dosage at DNA lesions (Gibbs-Seymour et al., 2015) (**Figure 2B**).

An obvious follow-up question to this study is: why is it so important to precisely regulate ID complex dosage at DNA damage sites by this elaborate mechanism? The authors suggest that this regulation by ubiquitin-SUMO crosstalk could help prevent excessive nucleolytic processing of DNA, downstream of ID complex monoubiquitylation. Like USP1-mediated deubiquination, SUMOylation of ID proteins could also be an important mechanism allowing for ID complex recycling and FA pathway regeneration. Future work will be needed to further understand mechanistically how dysregulation of ID complex dosage at DNA damage sites contributes to genome instability.

## SLX4 Acts as a SUMO E3 Ligase

One of the key steps in ICL repair is nucleolytic excision of the cross-link and downstream repair of the resulting DSB by HR. A key player that coordinates these complex repair processes is the SLX4 protein. Together with its activating subunit, SLX1, SLX4 associates with XPF-ERCC1 and MUS81-EME1 structure-specific endonucleases to cleave branched DNA structures (Andersen et al., 2009; Munoz et al., 2009; Stoepker et al., 2011). SLX4 additionally interacts with the mismatch repair proteins MSH2-MSH3 and is required for telomere stability through association with TRF2 (Wan et al., 2013). SLX1-SLX4 complexes serve as HJ resolvases and process multiple recombination intermediates (Fekairi et al., 2009). Thus, SLX4 serves as a scaffold for many different nucleases involved in ICL and HR repair, but our understanding of how these interactions are orchestrated to direct specific repair outcomes has been limited.

New studies from the Gaillard, Vertegaal, and Zou labs have shed light on SLX4 regulation, showing that SUMO and ubiquitin modifications are involved in directing the different activities of the SLX4 complex. Guervilly et al. (2015) made the surprising discovery that the SLX4 complex is a SUMO E3 ligase that SUMOylates SLX4 itself. SUMOylation by SLX4 is dependent on an interaction with the charged UBC9∼SUMO E2 enzyme as well as newly identified SIMs in SLX4. Studies from the Vertegaal lab identified three such SIMs in the SLX4 protein and showed that these motifs were critical for proper ICL repair and targeting of SLX4 to PML nuclear bodies and laser-induced DNA damage sites (González-Prieto et al., 2015). In addition to SUMOylating itself, SLX4 also targets the XPF subunit of the repair endonuclease XPF-ERCC1 for SUMO modification (Guervilly et al., 2015). Unexpectedly, the BTB domain of SLX4, which facilitates XPF targeting (Andersen et al., 2009), was specifically required for SUMOylation of XPF *in vivo* and *in vitro*, showing an additional role for the BTB domain outside of XPF binding (**Figure 2C**). At one extreme, chronic overexpression of SLX4 induces global replication stress and is extremely cytotoxic, which the authors suggest are consequences of extensive nucleolytic processing and chromatid breakage. On the other hand, the SUMO E3 ligase activity of SLX4 facilitates expression of common fragile sites (CFS), unstable genomic loci that are difficult to replicate. In fact, failure to localize SLX4 to CFS is associated with increased anaphase bridges and mitotic catastrophe. Therefore, on a global scale, increased SUMO ligase activity of SLX4 is detrimental as it contributes to replication stress, but is necessary to prevent mitotic catastrophe following CFS expression (Guervilly et al., 2015).

Ouyang et al. (2015) made similar findings about the involvement of SUMOylation in SLX4 activity. Namely, they also discovered that SLX4 binds SUMO2/3 chains via SIMs in a manner dependent on charged UBC9, and that SUMO interactions are important for suppressing fragile site instability and processing of CPT-induced replication intermediates. However, the authors extend these findings by directly comparing SLX4 targeting via its ubiquitin-binding zinc finger (UBZ) domains versus its SIM domains. While the UBZs of SLX4 are critical in ICL repair, the SIMs of SLX4 are instead more important for binding DNA damage sensors such as RPA, the MRN complex, and the telomere binding protein TRF2. Thus, the UBZs and SIMs of SLX4 are functionally distinct, providing a mechanism for targeting SLX4 in different repair contexts (Ouyang et al., 2015).

## Role of SUMOylated PCNA in Mammalian Cells

The FA pathway is important for activation of HR-mediated repair, a DSB repair mechanism that uses an intact sister chromatid as a repair template (review in Moynahan and Jasin, 2010). While HR has the advantage of being an error-free mode of repair as opposed to the non-homologous end joining (NHEJ) pathway, inappropriate hyperrecombination is associated with genomic instability and cancer (Martin et al., 2007). How the FA pathway coordinates crosslink repair and HR appropriately has been a poorly understood topic. It is known that in budding yeast, the UvrD domain helicase Srs2 limits inappropriate HR by removing RAD51 nucleofilaments from ssDNA at an early step in the HR pathway (Papouli et al., 2005). A homolog for Srs2 with similar antirecombinase activity had not been identified in human cells, however, prompting a recent study by the D’Andrea lab to identify novel HR regulators in mammalian cells. The authors identified C12orf48 as an anti-recombinase in human and chicken DT40 cells that specifically interacts with a PCNA-SUMO fusion protein *in vitro*. They named this protein PCNA associated recombination inhibitor (PARI). Similar to Srs2 in yeast, PARI restricts recombination by interfering with RAD51-DNA HR structures and is required for genome stability. Interestingly, PARI knockdown in FA cells, which are deficient in HR (Nakanishi et al., 2005), improves genome instability by increasing recombination frequency (Moldovan et al., 2012). These results indicate that manipulation of PARI levels could be an effective chemoprotective approach in HR-deficient cancers. Nevertheless, several key questions still remain pertaining to the role and recruitment of PARI to the replication fork, including whether SUMOylation of mammalian PCNA exists and whether PARI is truly the long sought-after functional human homolog of budding yeast Srs2.

## Role of SUMOylation in HR Repair

Following unhooking of an ICL by nucleolytic incisions, a DSB is generated that must be repaired by HR. Current evidence demonstrates a role for SUMOylation in coordinating this DSB repair step. SUMO E3 ligases PIAS1 and PIAS4 accumulate at DNA damage sites in mammalian cells, where they target multiple substrates for SUMO modification, including 53BP1 and BRCA1, to damage foci. SUMOylation facilitates the localization of these DNA repair proteins to DSB sites and also is required for RNF8 and RNF168-mediated ubiquitylation of target proteins (including H2A/X) to signal downstream repair (Galanty et al., 2009). In particular, SUMOylation of BRCA1 by PIAS SUMO E3 ligases increases its ubiquitin ligase activity, identifying it as a STUbL (Morris et al., 2009). In addition to BRCA1, another STUbL, RNF4, was identified that promotes DSB repair by regulating the turnover of DSB-responsive factors MDC1 and replication protein A (RPA), allowing for recruitment of factors necessary for DSB repair by HR (Galanty et al., 2012). Through the combination of these mechanisms (**Figure 2D**), SUMO-ubiquitin crosstalk amplifies DSB signaling to promote efficient DSB repair. Although likely, it is currently unclear whether regulation of BRCA1 by SUMOylation plays a role in the repair of ICLs as part of the Fanconi Anemia pathway.

## Role of SUMO-Like Domains (SLDs) in Dub-Substrate Interactions

While the importance of USP1/UAF1-mediated deubiquitylation of FANCD2 has been clearly established (Nijman et al., 2005; Oestergaard et al., 2007; Kim et al., 2009), the question of how USP1/UAF1 is targeted to the FANCI/FANCD2 heterodimer has remained elusive until recently. Yang et al. (2011) discovered that this targeting mechanism involves SUMO-like domains (SLD1 and SLD2) at the C-terminus of UAF1, which bind directly to SIM motifs of FANCI. Likewise, the SLD2 domain of UAF1 also binds to the SIM on hELG1 to direct USP1/UAF1 binding to another important substrate, PCNA-Ub (Huang et al., 2006). This SLD-SIM interaction is critical for FA pathway function, as deletion of the SLD2 sequence of UAF1 or the SIM of FANCI leads to deficient FANCD2 monoubiquitylation and DNA repair. Thus, SLD-SIM interactions provide a means for the regulated delivery of USP1/UAF1 DUB complex to its substrates for efficient ICL repair (Yang et al., 2011) (**Figure 2E**). As UAF1 is a highly abundant protein with many diverse binding partners, this study also points to the possibility that SLD-SIM targeting may also play broader roles not only in DNA repair, but as a general means to sort intracellular proteins involved in other processes.

## Future Perspectives

In summary, the above studies clearly demonstrate the importance of SUMO and SUMO-like modifications in fine-tuning FA pathway activation and DNA repair (summarized in **Figure 2**). It is probable that findings reported here represent a small portion of targets controlled by SUMOylation in the FA pathway, and future work should uncover other unidentified substrates and SUMO ligases/proteases critical to this process. These studies also point to the possibility of pharmacologically targeting SUMO ligases, SIM-SUMO interactions, and SENPs as a way to manipulate FA pathway activity in FA cells and HR-defective cancers. Consistent with this notion, several studies have reported the overexpression of SENPs in various disease conditions and cancers, prompting recent advancements in the development of small molecule inhibitors of SENPs with therapeutic potential (review in Kumar and Zhang, 2015). On the other hand, aggressive modulation of SUMO-ubiquitin signaling could also pose a risk for inefficient repair by the FA pathway and other DNA repair mechanisms. Thus, future studies are highly necessary to further understand the proper balance of SUMOylation and ubiquitination activity necessary for proper FA pathway function, and how dysregulation of these ubiquitin and ubiquitin-like post-translational modifiers underlies genome instability.

## Author Contributions

KC wrote the manuscript with advice from TH.

## Conflict of Interest Statement

The authors declare that the research was conducted in the absence of any commercial or financial relationships that could be construed as a potential conflict of interest.
